# Preconception leisure-time physical activity and family history of stroke and myocardial infarction associate with preterm delivery: findings from a Norwegian cohort

**DOI:** 10.1186/s12884-022-04528-y

**Published:** 2022-04-20

**Authors:** Tone Engen, Katrine Mari Owe, Julie Horn, Gerhard Sulo, Øyvind Erik Næss, Petur Benedikt Juliusson, Nils-Halvdan Morken, Grace Margrethe Egeland

**Affiliations:** 1 Department of Health and Caring Sciences, Western Norway University of Applied Sciences, Inndalsveien 28, 5063 Bergen, Norway; 2 Norwegian Research Centre for Women’s Health, Oslo University Hospital HF, Riks Hospital, Oslo, Norway and Division of Mental and Physical Health, Norwegian Institute of Public health, Oslo, Norway; 3 Department of Public Health and Nursing, NTNU, Norwegian University of Science and Technology, Trondheim, Norway & Department of Obstetrics and Gynecology, Levanger Hospital, Nord-Trøndelag Hospital Trust, Levanger Hospital, Kirkegata 2, N-7600 Levanger, Norway; 4 Centre for Disease Burden, Division of Mental and Physical Health, Norwegian Institute of Public Health, Bergen, Norway; & Oral Health Centre of Expertise in Western Norway-Vestland, Bergen, Norway; 5 Division of Mental and Physical Health, Norwegian Institute of Public Health & University of Oslo, Oslo, Norway &Institute of Health and Society, Faculty of Medicine, University of Oslo, Oslo, Norway; 6 Health Registries Research and Development, Division of Health Data and Digitalization, Norwegian Institute of Public Health, Bergen, Post Box 973 Sentrum, 5808 Bergen, Norway; 7 Department of Clinical Science, University of Bergen, Bergen, Norway; 8 Department of Obstetrics and Gynecology, Haukeland University Hospital, Bergen, Norway & Centre for Fertility and Health, Norwegian Institute of Public Health, Oslo, Norway; 9 Department of Global Public Health and Primary Care, University of Bergen, Bergen, Norway

**Keywords:** Preterm birth, Pregnancy, Cardiovascular disease, Leisure time physical activity, women’s health, Cohort study

## Abstract

**Background:**

Preterm birth poses short and long-term health consequences for mothers and offspring including cardiovascular disease sequelae. However, studies evaluating preexisting family history of cardiovascular disease and risk factors, such as physical activity, as they relate prospectively to risk of delivering preterm are lacking.

**Objectives:**

To evaluate whether preconception past-year weekly leisure-time physical activity or a family history of stroke or of myocardical infarction prior to age 60 years in first degree relatives associated, prospectively, with preterm delivery.

**Design:**

Cohort study. Baseline data from Cohort Norway (1994–2003) health surveys were linked to the Medical Birth Registry of Norway for identification of all subsequent births (1994–2012). Logistic regression models provided odds ratios (OR) and 95% confidence intervals (CI) for preterm delivery (< 37 weeks gestation); multinomial logistic regression provided OR for early preterm (< 34 weeks) and late preterm (34 through to end of 36 weeks gestation) relative to term deliveries.

**Results:**

Mean (SD) length of time from baseline health survey participation to delivery was 5.6 (3.5) years. A family history of stroke associated with a 62% greater risk for late preterm deliveries (OR 1.62; CI 1.07–2.47), while a family history of myocardial infarction associated with a 66% greater risk of early preterm deliveries (OR 1.66; CI 1.11–2.49). Sensitivity analyses, removing pregnancies complicated by hypertensive disorders of pregnancy, diabetes mellitus, and stillbirth deliveries, gave similar results. Preconception vigorous physical activity of three or more hours relative to less than 1 h per week associated with increased risk of early preterm delivery (OR 1.52; 95% CI 1.01–2.30), but not late or total preterm deliveries. Light physical activity of three or more hours per week relative to less activity prior to pregnancy was not associated with early, late, or total preterm deliveries.

**Conclusions:**

Results suggest that family history of cardiovascular disease may help identify women at risk for preterm delivery. Further, research is needed regarding preconception and very early pregnancy vigorous physical activity and associated risks.

## Introduction

Preterm birth, defined as birth prior to 37 weeks gestation, affects 5–13% of births in developed countries, and proportions of preterm births are increasing for multiple and singleton births alike [1]. While advances in perinatal medicine have contributed to increased survival rates for preterm births, preterm deliveries remain the greatest current challenge in perinatal medicine [2]. Being born prematurely is a significant predictor of a wide range of short and long-term health-related problems [3], including cardiovascular and renal sequelae [2]. Also, for mothers a preterm delivery associates with an increased risk of cardiovascular disease later in life (CVD) [4–6].

Prematurity is considered part of the “great obstetrical syndrome”, a collective designation for a variety of pregnancy complications that may share the same pathological pathways related to disorders of placentation and placental development [7, 8]. Interestingly, many of these obstetrical syndrome complications also associate with increased future risk of CVD in women [4–6, 9].

Among the modifiable risk factors for CVD, leisure-time physical activity (LTPA) is a recommended and effective means of reducing CVD risk. The British National Health Service (NHS) recommends pregnant women to maintain a daily activity level equal to their pre-pregnancy routine, but, as a general rule, not to become breathless during exercise [10, 11]. Similar recommendations are also provided by the American College of Obstetrics and Gynecology (ACOG) and World Health Organization (WHO) for pregnant women without contra-indications for physical activity, [12, 13]. Studies have noted potential adverse effects of participation in vigorous physical activity during pregnancy [14–16], and have also raised questions regarding recommendations on LTPA for obese women [17].

While numerous risk factors for preterm deliveries have now been established [18], there has been a lack of research evaluating maternal pre-pregnancy modifiable and non-modifiable factors such as physical activity patterns and family history of CVD for their association with preterm deliveries. Therefore, the aim of the current study was to separately evaluate the association of LTPA and family history of CVD prior to pregnancy for their association with preterm delivery in a prospective population-based cohort of reproductive age women in Norway.

## Methods

Women participating in Cohort Norway (CONOR) health surveys conducted between 1994 and 2003 [19] were linked to the Medical Birth Registry of Norway (MBRN) [20] for identification of all subsequent deliveries through to the end of 2012 using a unique identifier replaced by a project-specific pseudo-id for the women: details of which are presented elsewhere [21]. Participants were also linked to pregnancies prior to CONOR participation for ascertainment of a prior preterm delivery among parous women. Due to age differences in recruitment to the health surveys, data for the current analyses came predominately from three CONOR surveys which included women of reproductive age: The HUBRO Study in Oslo (24%), The Tromsø Study (21%), and the Nord-Trøndelag Health Study (HUNT, 50%), and the remaining from diverse survey regions. The HUBRO Study in Oslo oversampled immigrants: 38% of the HUBRO Study participants were not born in Norway, otherwise the study sample primarily represents ethnic Norwegians.

We utilized CONOR questionnaires for descriptive baseline self-reported background information of mothers. The a-priori attributes of primary interest included self-reported frequency of past-year weekly light and vigorous LTPA and family history of stroke or of a myocardial infarction younger than 60 years of age in siblings and/or parents (first degree relatives). LTPA questions had four answer options (none, < 1 h., 1–2 h., and 3 or more hrs. per week). Vigorous activity was defined as any activity resulting in sweating and/or shortness of breath, whereas light activity was defined by the absence of sweating and/or shortness of breath. Vigorous LTPA was combined into three categories (< 1 h, 1–2 h and ≥ 3 h per week), whereas light LTPA was combined into two groups (< 3 h and ≥ 3 h per week) given that 1–2 h of light LTPA per week is below recommended activity levels.

CONOR also assessed baseline self-reported characteristics including health status (diabetes mellitus, chronic hypertension, and asthma), educational level (primary, secondary, or any college/university), daily cigarette smoking (yes vs. no), and marital status (married/cohabitation vs. other). Measurements including weight and height for calculating body mass index (BMI, kg/m^2^) and resting systolic and diastolic blood pressure were taken by trained personnel as part of the baseline CONOR assessments.

### Medical birth registry of Norway (MBRN)

The MBRN is based on compulsory notification of all live- and stillbirths from week 16 of gestation through standardized forms completed by obstetric nurse midwife/birth attendants since 1967 [20]. The MBRN provided data regarding gestational age, birth weight, and numerous medical risk factors for or associates of preterm delivery: pre-pregnancy thyroid condition, chronic hypertension, early and mid-pregnancy bleeding, placental abruption or previa, preeclampsia/eclampsia (PE), gestational hypertension, pregestational and gestational diabetes, congenital malformations and stillbirth deliveries, and whether pregnancy was a result of assisted reproductive technology (ART). Identification of preterm deliveries (early preterm < 34 weeks of gestation; late preterm ≥34 <  37 weeks of gestation) were based upon gestational age predicted by ultrasound when available. In this study, 17% of deliveries had missing data on gestational age by ultrasound. Thus, for these deliveries, we used date of last menstrual period to estimate gestational age.

### Exclusions

**Fig. 1:**
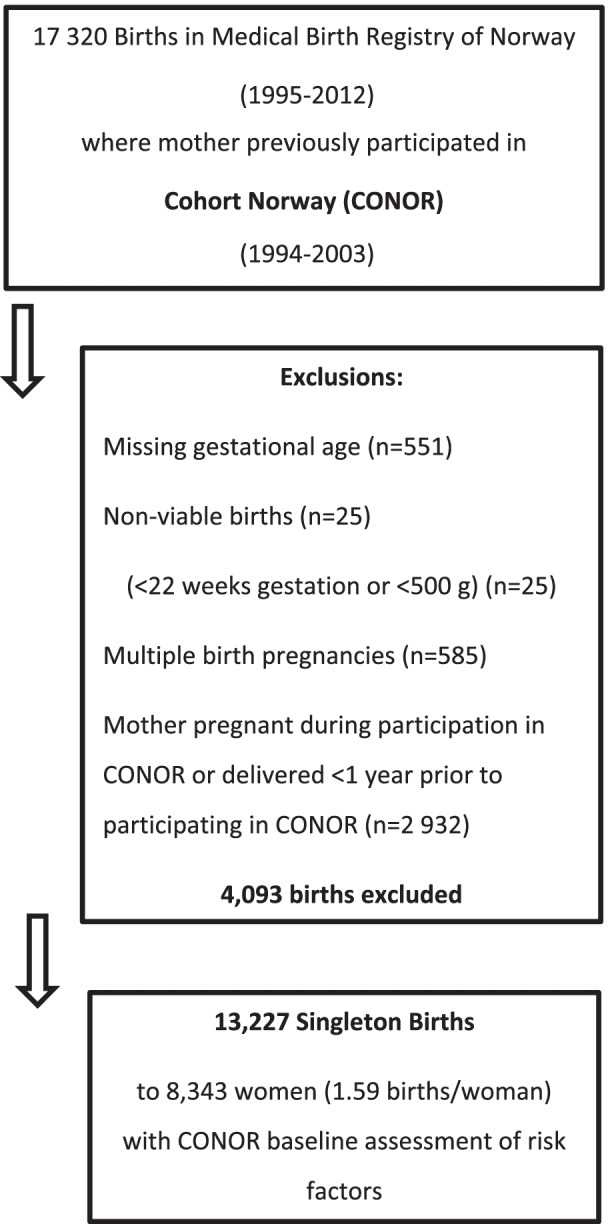
Flowchart of the selection of the current study sample from Cohort Norway (CONOR)

We identified 17,320 births registered in the MBRN where the mother had participated in a CONOR health survey prior to delivery (Fig. 1). We excluded births missing gestational age, non-viable births (birth weight < 500 g and/or < 22 weeks of gestation), and multiple birth pregnancies. We also excluded deliveries if mother was pregnant while participating in CONOR survey or if she was pregnant in the year prior to CONOR participation. After exclusions, we had a total of 13,227 eligible births to 8343 women.

### Statistical analyses

Cohort descriptive characteristics are reported as percent or mean (SD). Multivariable logistic regression evaluated odds ratios (OR) and 95% confidence intervals (CI) for preterm delivery (< 37 weeks gestation), while multinomial logistic regression evaluated odds ratios (OR) for early preterm (< 34 weeks) and late preterm (34 through to end of 36 completed weeks gestation) relative to term deliveries. Births were the unit of analyses where mothers’ pseudo-id was entered as a cluster variable in regression analyses. There were four independent variables of interest: family history of stroke, family history of myocardial infarction prior to age 60 years, light LTPA and vigorous LTPA. Multivariable models considered important covariates. Model 1 included baseline age, length of time between CONOR participation and delivery, and parity risk category (nulliparous, parous with a prior preterm delivery, and the referent group of parous women with no prior preterm delivery). Model 2 included Model 1 covariates and baseline education (primary, secondary, or any college/university), daily cigarette smoking (yes vs. no), height (cm), and BMI status (underweight < 18.5, obese > 30, vs. referent group BMI of 18.5–29.9 kg/m^2^). Overweight (25–29.9 kg/m^2^) and normal weight (18.5–24.9 kg/m^2^) women were combined into one referent group as initial analyses showed no excess risk of premature deliveries to overweight relative to normal weight women. In the evaluation of LTPA, model 3 included model 2 covariates and both light and vigorous LTPA.

Supplementary analyses considered Model 3 covariates with medical conditions considered risk factors for or associates of preterm delivery: recipient of ART, a pre-pregnancy thyroid condition, one or more of the following three conditions combined (early or mid-pregnancy bleeding, placental abruption, or placenta previa), any congenital malformation, chronic hypertension and diabetes mellitus noted in CONOR health surveys and/or the MBRN, PE, or stillbirth deliveries. Further, supplemental analyses considered family history of stroke and myocardial infarction together with LTPA with model 3 covariates. Asthma reported in the baseline health surveys was not related to early, late or total preterm deliveries and was therefore not considered in these supplementary analyses.

A sensitivity analysis was conducted removing those with stillbirth delivery, chronic hypertension, PE, or diabetes (*n* = 920) as these conditions were considered potential intermediaries between physical activity and preterm delivery and between family histories and preterm deliveries. Also, as parous and nulliparous women may have different physical activity patterns, we conducted an additional sensitivity analyses of physical activity limited to nulliparous women at baseline. Finally, we conducted a sensitivity analysis removing those with a family history of stroke or of myocardial infarction.

While the Oslo region included immigrant women, we lacked data on immigrant status of participants for our analyses. Survey regions did not relate to risk of preterm or to LTPA patterns and was therefore not considered as a covariate in the analyses. However, while the mean and median heights were similar between the three survey regions, the Oslo region had a wider SD in heights likely reflecting the greater variability in women’s heights associated with a population which includes immigrant women from countries with shorter heights than that of Western Europe and Norway [22]. The inclusion of height in our multivariable models was a means of adjusting, albeit indirectly, for immigrant status in addition to adjusting for the strong inverse association overall between maternal height and preterm delivery.

Data on light LTPA was missing for 6.0% (*n* = 788), and vigorous LTPA for 6.4% (*n* = 840) of participants. Otherwise, the percent missing data was low for most covariates: BMI, height, education (< 1.5%), and smoking (4.7%). Analyses were based upon complete data available for the independent variables and covariates. Statistical analysis was performed using Stata version 15 (StataCorp LP, 2017, College Station, TX, USA).

## Results

There was a total of 13,227 births representing 8343 women with an average of 1.59 births per woman. The mean age (SD) of women at the time of participation in the baseline survey was 27.9 (4.5) years, 24.3% had attained any college or university education, 6.7% were obese, 26.9% smoked cigarettes daily, and 48.9% engaged in light LTPA while 16.9% engaged in vigorous LTPA of 3 h or more per week in the past year (Table 1). Deliveries following ART represented 3% of births. We identified 718 premature deliveries (5.4%) in the dataset: 209 deliveries were early preterm, and 509 deliveries were late preterm.

**Table 1:** Descriptive characteristics of mothers: Cohort Norway and. Medical Birth Registry of Norway (*N* = 13,227)

Baseline Cohort Norway Characteristics	Mean (SD)^a^
Age (years)	27.9 (4.5)
BMI (kg/m^2^)	23.9 (3.8)
Obese (> 30 kg/m^2^), %	6.7
Height (cm)	166.3 (6.5)
High blood pressure, %	4.6
Smoking cigarettes daily, %	26.9
Light physical activity, > 3 h/wk., %	48.9
Vigorous physical activity, > 3 h/wk., %	16.9
Region, Oslo	25.0
Any college/university, %	24.3
Time to delivery (years)	5.6 (3.5)
**Medical Birth Registry of Norway Characteristics**	
Nulliparous, %	29.9
Preeclampsia or gestational hypertension, %	5.3
Diabetes mellitus (pregestational, gestational, type unspecified)^b^	1.7
Preterm (< 37 weeks of gestation), %	5.4
Prior preterm, %	1.8

^a^Unless noted as percent, %

^b^Includes diabetes mellitus also noted in Cohort Norway

### Family history of stroke and myocardial infarction

A family history of stroke associated with an increased risk of preterm deliveries in model 1 and model 2 analyses while a family history of myocardial infarction did not associate with risk of preterm deliveries (Table 2). In supplemental multivariable analyses including medical conditions that are known risk factors for preterm delivery, a family history of stroke remained a significant predictor of preterm delivery. Further in sensitivity analyses removing those with either chronic hypertension, preeclampsia, diabetes mellitus, and stillbirth deliveries, a family history of stroke remained a significant predictor of preterm delivery (model 2 OR of 1.60; 95% CI 1.05–2.45).

**Table 2:** Family history of stroke and myocardial infarction < 60 years and risk of preterm delivery

		Preterm (< 37 weeks gestation), *n* = 718			Early Preterm (< 34 weeks), *n* = 209		Late Preterm (34–36 weeks), *n* = 509	
	N	(%)	OR (95% CI)^a^	OR (95% CI)^a^	OR (95% CI)^b^	OR (95% CI)^b^	OR (95% CI)^b^	OR (95% CI)^b^
Family history			Model 1^c^	Model 2^d^	Model 1^c^	Model 2^d^	Model 1^c^	Model 2^d^
Stroke								
No	12,672	(5.3)	1.0	1.0	1.0	1.0	1.0	1.0
Yes	555	(8.5)	1.60 (1.11–2.30)	1.56 (1.07–2.27)	1.46 (0.82–2.60)	1.41 (0.77–2.57)	1.64 (1.09–2.48)	1.62 (1.07–2.47)
Myocardial infarct < 60 yrs.								
No	12,115	(5.3)	1.0	1.0	1.0	1.0	1.0	1.0
Yes	1112	(6.5)	1.19 (0.90–1.56)	1.15 (0.87–1.53)	1.71 (1.15–2.54)	1.66 (1.11–2.49)	1.00 (0.72–1.39)	(0.68–1.36)

*CI* Confidence intervals, *OR* Odds ratio

^a^Logistic regression models entering mothers’ pseudo-ID as cluster variable.

^b^Mlogit models; mothers’ pseudo-ID entered as cluster variable.

^c^Model 1 includes Cohort Norway baseline age and time to delivery, and parity risk (nulliparous, prior preterm among parous, and parous without prior preterm delivery).

^d^Model 2 includes model 1 covariates and height (cm), body mass index status (kg/m^2^; < 18.5; 18.5–29.9, and > 30), daily cigarette smoking (yes vs. no), educational level (primary, high school/vocational school, any college/university).

In analyses evaluating early and late preterm deliveries, the excess risk associated with a family history of stroke was strongest and statistically significant for late preterm deliveries where we observed a 62% elevated risk of late preterm associated with a family history of stroke (model 2). In contrast, a family history of myocardial infarction associated with a 66% greater risk of early preterm deliveries (model 2: OR of 1.66; 95% CI 1.11–2.49). Additional adjustment for known medical risk factors for preterm delivery did not alter the results presented for family history of stroke or myocardial infarction. Similarly, in the sensitivity analyses removing chronic hypertension, preeclampsia, diabetes mellitus, and stillbirth deliveries, results were similar for a family history of stroke for early preterm delivery (model 2 OR 1.47; 95% CI 0.81–2.68) and for late preterm delivery (model 2 OR 1.74; 95% CI 1.14–2.65). Further, a family history of myocardial infarction prior to age 60 years remained a significant associate of early preterm delivery (model 2 OR 1.77; 95% CI 1.18–2.65), but not for late preterm delivery (model 2 OR 1.04; 95% CI 0.74–1.46).

### Light and vigorous leisure-time physical activity

Light LTPA did not associate with early, late or total preterm delivery in any analyses (Tables 3 & 4). For vigorous physical activity, we observed no association with total preterm delivery. However, we did observe a 52% excess risk for early preterm delivery associated with 3 h or more of weekly vigorous physical activity compared to less than 1 h of vigorous LTPA (model 2, OR of 1.52; 1.01–2.30) (Table 4). The association between vigorous LTPA and early preterm delivery became stronger in the analyses adjusting for light LTPA (Table 4; model 3). Results were similar in further analyses adjusting for known medical risk factors for preterm delivery and were similar in an additional model adjusting for family history of stoke or of myocardial infarction.

**Table 3:** Preconception light and vigorous past-year weekly physical activity and preterm delivery

		Preterm (< 37 weeks gestation), *n* = 718			
Past-year	N	(%)	OR (95% CI)^a^	OR (95% CI) ^a^	OR (95% CI) ^a^
			Model 1^b^	Model 2^c^	Model 3^d^
Light LTPA					
< 3 h/wk	6365	5.7	1.0	1.0	1.0
> 3 h/wk	6074	5.2	0.88 (0.74–1.04)	0.94 (0.78–1.12)	0.86 (0.71–1.04)
Vigorous LTPA					
None	6426	5.4	1.0	1.0	1.0
1–2	3865	5.2	0.96 (0.79–1.16)	1.04 (0.85–1.27)	1.06 (0.85–1.31)
> 3 h/wk	2096	5.5	1.03 (0.81–1.31)	1.17 (0.91–1.50)	1.31 (1.00–1.71)

*CI* Confidence intervals, *LTPA* Leisture-time physical activity, *OR* Odds ratio, *wk* Week

^a^Logisticregression models entering mothers’ pseudo-ID as cluster variable.

^b^Model 1 includes Cohort Norway baseline age and time to delivery, and parity risk (nulliparous, prior preterm among parous, and parous without prior preterm delivery).

^c^Model 2 includes model 1 covariates, and height (cm), body mass index (kg/m^2^) status (underweight: < 18.5; normal/overweight: 18.5–29.9, and obese), daily cigarette smoking (yes vs. no), educational level (primary, high school/vocational school, any college/university).

^d^Model 3 includes model 2 covariates and simultaneously light and vigorous leisure-time physical activity.

**Table 4:** Preconception light and vigorous past-year leisure-time physical activity and early and late preterm delivery

Past-year	Early Preterm (< 34 weeks), ***n*** = 209			Late Preterm (34–36 weeks), ***n*** = 509		
	OR (95% CI)^a^	OR (95% CI)^a^	OR (95% CI)^a^	OR (95% CI)^a^	OR (95% CI)^a^	OR (95% CI)^a^
	Model 1^b^	Model 2^c^	Model 3^d^	Model 1^b^	Model 2^c^	Model 3^d^
Light LTPA						
< 3 h/week	1.0	1.0	1.0	1.0	1.0	1.0
> 3 h/week	0.80 (0.60–1.07)	0.94 (0.70–1.27)	0.79 (0.57–1.09)	0.91 (0.75–1.11)	0.94 (0.76–1.16)	0.89 (0.71–1.11)
Vigorous LTPA						
None	1.0	1.0	1.0	1.0	1.0	1.0
1–2 h/week	0.91 (0.64–1.28)	1.06 (0.74–1.51)	1.15 (0.78–1.70)	0.96 (0.77–1.20)	1.02 (0.81–1.29)	1.02 (0.80–1.30)
> 3 h/week	1.21 (0.81–1.80)	1.52 (1.01–2.30)	1.92 (1.24–2.97)	0.95 (0.72–1.25)	1.04 (0.77–1.39)	1.11 (0.81–1.51)

*CI* Confidence intervals, *LTPA* Leisure-time physical activity, *OR* Odds ratio

^a^Mlogit models; mothers’ pseudo-ID entered as cluster variable.

^b^Model 1 includes Cohort Norway baseline age and time to delivery, and parity risk (nulliparous, prior preterm among parous, and parous without prior preterm delivery).

^c^Model 2 includes model 1 covariates, and height (cm), body mass index (kg/m^2^) status (underweight: < 18.5; normal/overweight: 18.5–29.9, and obese), daily cigarette smoking (yes vs. no), educational level (primary, high school/vocational school, any college/university).

^d^Model 3 includes model 2 covariates and simultaneously light and vigorous leisure-time physical activity

### Sensitivity analyses

In sensitivity analyses removing intermediaries (stillbirth deliveries, chronic hypertension, preeclampsia, and diabetes mellitus), we observed a similar OR for early preterm delivery associated with 3 h or more of vigorous activity vs. less than 1 h per week but with wider CI (model 2, OR 1.57; 95% CI of 0.95–2.60, *p*-value = 0.08) (data not shown). Further, in the sensitivity analyses limited to nulliparous women at baseline, the excess risk of early preterm delivery associated with three or more hours of vigorous LTPA remained statistically significant where we observed more than a 2-fold increased risk of early preterm delivery (model 3, OR 2.17; 95% CI 1.18–4.01) (data not shown). Further, these findings remained significant after removing the intermediaries. Also, results similar with our primary analyses were obtained in the analyses removing those with a family history of stroke or myocardial infarction where vigorous LTPA of 3 or more hours per week associated with an 88% increased risk of early preterm delivery vs. less than 1 h per week (model 3, OR 1.88; 95% CI of 1.16–3.04) (data not shown). Similar with the primary analyses, light LTPA of 3 or more hours per week relative to less activity was not significantly related to risk of total preterm, early, or late preterm delivery in any of the models among women who were nulliparous at baseline (data not shown), or among those without a family history of stroke or myocardial infarction.

## Discussion

We observed an increased risk of early preterm deliveries in women with a family history of myocardial infarction prior to age 60 years, and an increased risk of preterm births overall and of late preterm births in women with a family history of stroke. Past-year vigorous LTPA of three or more hours per week associated with a 50% increased risk of early preterm delivery compared to less than 1 h of vigorous LTPA per week in our primary analyses. Preconception vigorous LTPA remained a significant predictor of early preterm delivery in supplementary analyses adjusting for known risk factors for preterm delivery and in the sensitivity analyses.

### Family history of stroke and myocardial infarction

Our findings that a family history of stroke and MI predicted preterm delivery adds importantly to the existing literature. Previous studies have focused on the subsequent risk of CVD outcomes among women with preterm deliveries [4–6]. For example, Poorthuis [6] reported that women with a previous preterm delivery had an 86% greater risk of stroke (ischemic or hemorrhagic; pooled OR of 1.86; 95% CI 1.15–3.02) compared to women with no pregnancy complication. In a systematic review, Grandi and colleagues [5] concluded that women with certain pregnancy complications, including preterm birth, require routine life-long follow-up health screenings and medical care given their increased risk for future CVD. Our findings, however, suggests that screening for a family history of CVD prior to or in early pregnancy would help identify women at higher risk of preterm birth.

Multiple pathways have been suggested by which underlying CVD mechanisms may place women at risk for preterm delivery and could contribute to the association we observed between a family history of stroke and MI and greater risk for preterm delivery. Factors associated with a family history of CVD may relate to suboptimal functioning of the vascular bed and inadequate placentation to support fetal growth and development. Placental dysfunction due to poor placentation is a contributing risk factor for preterm births [23]. A recent study of lipid levels and immune- and inflammatory biomarkers including cytokines and chemokines, identified several subgroups of pregnancies at risk for placental dysfunction associated with premature birth [24]. Likewise, an inflammatory phenotype is associated with cardiovascular disease in adults [9]. Further, women’s normal physiological response to pregnancy involves increases in lipids, coagulation, insulin resistance, and blood pressure: all risk factors for CVD. However, for women with underlying CVD risk, the physiological responses characteristic of pregnancy may increase risk of pregnancy-related complications [25].

### Leisure-time physical activity

Our finding that vigorous LTPA of at least 3 h per week prior to pregnancy associated with an increased risk of early preterm delivery, independent of stillbirth, preeclampsia, and other high-risk pregnancies, adds importantly to the literature. None of the previous studies evaluating preterm delivery risk by LTPA levels have specifically evaluated vigorous intensity LTPA prior to pregnancy for its association with risk of early preterm delivery. Given that pre-pregnancy physical activity is the strongest predictor of physical activity in pregnancy and that the level of activity declines during pregnancy [26, 27], it is possible that reported LTPA in our study reflects LTPA patterns very early in pregnancy. However, we would expect some degree of misclassification given the potential for changes in physical activity levels during the follow-up period and due to pregnancy-related symptoms such as vomiting and nausea.

While numerous studies have evaluated the relationship between LTPA during pregnancy and preterm delivery, sample sizes and the definitions of physical activity have varied greatly between studies. LTPA is most often assessed either after the first trimester or retrospectively within a day or more after delivery. Focusing on studies of at least 4000 participants, the preponderance of evidence suggests that engaging in LTPA during pregnancy is protective of preterm delivery [28–32]. A Danish cohort study of pregnant women completing 22 weeks of gestation in a singleton pregnancy, found a reduced risk of preterm birth in physically active women regardless of type of exercise (high vs. low impact) and metabolic equivalents of tasks (MET) scores compared to inactive women [28]. In the same Danish cohort, a reduced preterm risk was observed among women swimming or bicycling relative to sedentary women [29]. In another Danish study population, women reporting engaging in moderate to heavy physical activity at approximately 16 weeks gestation had a lower risk of preterm birth compared to sedentary women [30]. In Brazil, retrospective reports of engaging in any form of LTPA for at least 90 min per week during all three trimesters of pregnancy associated with lower risk of preterm deliveries relative to less active women [31]. In a cohort of 61,098 Norwegian women participating in the Norwegian Mother, Father, and Child Cohort Study, exercise up to five times per week (assessed at 17 weeks of gestation) associated with a lower risk of preterm birth while exercise of 6 or more times per week associated with a non-significant tendency for a higher risk of preterm birth [32]. In Wuhan region of China, a case-control study identified a potential U-shaped association between outdoor LTPA exercise in late pregnancy (in minutes per day reported after delivery) and risk of preterm birth where the benefits of physical activity relative to being sedentary were evident at up 150 min/day. The study noted an upswing in preterm risk at levels approaching and higher than 200 min/day, but with very wide CI given small numbers of very active women in the study [33].

There is evidence that high-intensity physical activity during pregnancy increases risk of fetal bradycardia and miscarriage [14, 15]. Also, in another study utilizing the same study population as the current study, preconception vigorous LTPA associated with an increased risk of stillbirth deliveries [16]. Further, recreational physical activity before pregnancy was associated with an increased risk of stillbirth in obese (BMI ≥30 kg/m^2^) pregnant women compared to normal weight women in a Norwegian study [17].

### Strengths and limitations

Strengths include the prospective study design in a large study population with complete ascertainment of subsequent pregnancies in the MBRN, which includes standardized collection of maternal and birth characteristics. The baseline survey and the MBRN data enabled consideration of a wide range of known risk factors for preterm delivery. Another strength is the assessment of LTPA before pregnancy which precludes the possibility of recall bias.

The study, however, also has limitations. While the physical activity questions have been validated [35], vigorous activity was based upon self-report and defined as activities resulting in sweating or shortness of breath. While self-rated exertion is an indirect measure of relative intensity of physical activity and widely used in the literature, exertion may vary by individual level of cardiovascular fitness and type of activities [36]. Also, we did not have information on type of activity. Thus, our assessments cannot precisely estimate the degree of intensity of vigorous LTPA. Further, we had no information regarding moderate intensity LTPA. Also, with a mean follow-up time of 5.6 years between CONOR participation and delivery, we cannot rule out that some women may have changed their lifestyle including their level of LTPA during this period. Importantly, pregnancy-related symptoms, such as nausea and vomiting, could influence LTPA during pregnancy. At 17 weeks gestation, women experiencing nausea and vomiting were 22% less likely to engage in regular exercise [32]. However, these sources of misclassification would tend to weaken and not strengthen the associations observed. Another limitation is that we lacked information on country region of birth. The study population, however, was largely ethnic Norwegian and we did adjust for height which co-associates with mothers’ country region of birth. Another limitation is that number of children in the home may have influenced women’s activity levels. We did adjust for parity and we conducted a sensitivity analyses limited to nulliparous women in which we observed similar results to our primary analyses. However, we had no information of partner’s children in the home.

## Conclusion

The findings from this study are of clinical significance given that a family history of CVD could help identify women in need of greater obstetric care. A careful selection of women in need of high-risk pregnancy follow-up decreases risk of potentially harmful, untimely, or unnecessary interventions in low-risk pregnancies, thus freeing resources for specialized care [37]. Our findings also suggest the need for more research to closely evaluate the potential risks associated with participating in vigorous physical activity immediately prior to and very early during pregnancy.

## Data Availability

The data that support the findings of this study are available from the Medical Birth Registry of Norway and Cohort Norway but restrictions apply to the availability of these data, which were used under license for the current study, and so are not publicly available. Data are however available if permissions and approvals are granted by the Norwegian Institute of Public Health, Regional Medical Research Ethics Committee and the Norwegian Data Protection Agency.

## Abbreviations

ACOG: American College of Obstetrics and Gynecology
ART: Assisted Reproductive Technology
BMI: Body Mass Index
CI: Confidence interval
CONOR: Cohort of Norway
LTPA: Leisure time physical activity
MBRN: The Medical Birth Registry of Norway
METS: Metabolic equivalent of tasks
MI: Myocardial infarction
NHS: National Health Service
OR: Odds ratio
SD: Standard deviation
WHO: World Health Organization
